# A Brief Review of Natural Products with Urate Transporter 1 Inhibition for the Treatment of Hyperuricemia

**DOI:** 10.1155/2022/5419890

**Published:** 2022-10-28

**Authors:** Qianghua Yuan, Yuan Cheng, Rong Sheng, Yan Yuan, Mei Hu

**Affiliations:** ^1^Hospital of Chengdu University of Traditional Chinese Medicine, Chengdu, Sichuan 610041, China; ^2^School of Medical and Life Sciences/Reproductive & Women-Children Hospital, Chengdu University of Traditional Chinese Medicine, Chengdu, Sichuan 610041, China

## Abstract

Hyperuricemia is a common disease caused by a high level of uric acid. Urate transporter 1 (URAT1) is an important protein and mediates approximately 90% of uric acid reabsorption. Therefore, the URAT1 inhibitor is a class of uricosuric medicines widely used in the clinic for the treatment of hyperuricemia. To find the new medicine with stronger URAT1 inhibition and lower toxicity, researchers have been exploring natural products. This study systematically summarizes the natural products with URAT1 inhibition. The results show that many natural products are potential URAT1 inhibitors, such as flavonoids, terpenoids, alkaloids, coumarins, stilbenes, and steroids, among which flavonoids are the most promising source of URAT1 inhibitors. It is worth noting that most studies have focused on finding natural products with inhibition of URAT1 and have not explored their activities and mechanisms toward URAT1. By reviewing the few existing studies of the structure-activity relationship and analyzing common features of natural products with URAT1 inhibition, we speculate that the rigid ring structure and negative charge may be the keys for natural products to produce URAT1 inhibition. In conclusion, natural products are potential URAT1 inhibitors, and exploring the mechanism of action and structure-activity relationship will be an important research direction in the future.

## 1. Introduction

Uric acid is the end metabolite derived from the oxidation of purine compounds [1]. Hyperuricemia is a chronic metabolic disease caused by a high level of uric acid. Excessive intake of purine-containing foods and insufficient uric acid excretion are the keys to causing hyperuricemia [2]. In recent years, the incidence of hyperuricemia has continued to increase worldwide, which may be related to changes in lifestyle, such as the prevalence of a high-purine diet, fructose beverages, and alcohol consumption [3, 4]. In China, the overall prevalence of hyperuricemia is 13.3%, and the prevalence in men is higher than in women [5]. In the United States, the prevalence of hyperuricemia is 21.2% in men and 21.6% in women [6]. Hyperuricemia is related to the occurrence of many diseases, such as cardiovascular disease, metabolic syndrome, and acute kidney injury [7]. Therefore, patients have an urgent need for efficient and safe therapeutic methods or drugs [8].

Reducing purine intake, inhibiting uric acid production, and promoting uric acid excretion are effective ways to treat or improve hyperuricemia [9]. URAT1 inhibitors are a widely used class of uricosuric drugs by inhibiting the reabsorption of uric acid, such as probenecid, sulfinpyrazone, and benzbromarone [10]. Although these drugs have good uric acid lowering effects, they all have varying degrees of side effects [11]. Currently, sulfinpyrazone has been withdrawn from most countries due to its severe gastrointestinal toxicity [12]. Benzbromarone has severe hepatotoxicity and is currently approved for use in only a few countries [13]. Even the newly approved lesinurad has renal toxicity and cardiovascular toxicity [14]. Therefore, scholars have been exploring new URAT1 inhibitors with low toxicity [15].

Natural products refer to components or metabolites from animals, plants, insects, and microorganisms, such as proteins, peptides, polysaccharides, and alkaloids [16–18]. Natural products have been used as medicines for thousands of years. Moreover, the importance of natural products is increasing day by day and has become an important source of drug development [19]. At present, long-term clinical practice has demonstrated that traditional Chinese medicine (one of the important sources of natural products) has exact efficacy in lowering serum uric acid without serious adverse effects [20]. With the deepening of research, scholars have found that natural products are expected to be the source of new URAT1 inhibitors. This study systematically summarizes natural products with URAT1 inhibition. The results showed that flavonoids, terpenoids, alkaloids, coumarins, stilbenes, steroids, organic acids, and polysaccharides show inhibitory effects of URAT1, which can inhibit URAT1 activity and promote uric acid excretion. However, most studies have focused on finding natural products with inhibition of URAT1 and have not explored their activities and mechanisms towards URAT1. By reviewing the few existing studies on the structure-activity relationship studies and analyzing common features of natural products with URAT1 inhibition, we speculate that the rigid ring structure and negative charge may be the keys for natural products to produce URAT1 inhibition. In conclusion, natural products are valuable sources of URAT1 inhibitor, and exploring the mechanism of action and structure-activity relationship will be an important research direction in the future.

## 2. Pathological Processes of Hyperuricemia and the Role of URAT1

**Figure 1:**
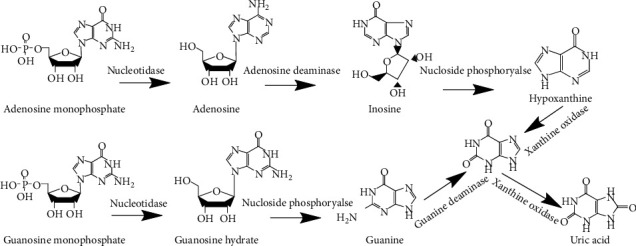
The production pathway of uric acid.

Uric acid, also known as 2,6,8-trihydroxypurine, is a heterocyclic carbonyl compound with a relative molecular weight of 168 [21]. Uric acid is mainly produced by the metabolism of endogenous and dietary purine compounds under the action of xanthine oxidase in the liver (Figure 1) [22]. Hyperuricemia refers to an excessively high concentration of uric acid in the blood. That is, uric acid concentration <7.0 mg/dl in men or <6.0 mg/dl in women [23]. As a metabolic disease, hyperuricemia is closely related to the occurrence and development of many diseases, such as gout, hypertension, heart disease, and diabetes [24]. The appearance of gout is most closely related to hyperuricemia. This is because an excessively high concentration of uric acid is easily deposited in the articular cavity in body tissue, causing pain, edema, and inflammation in the joints, finally inducing gout [25].

**Figure 2:**
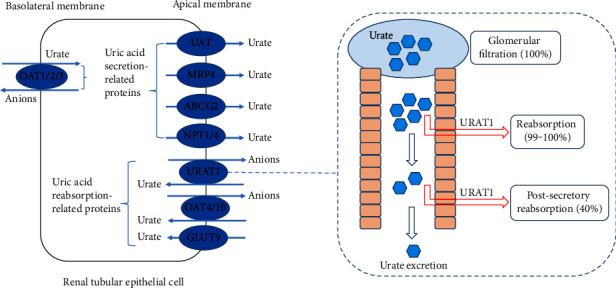
The distribution of uric acid transporters and the effects of URAT1 on uric acid metabolism.

The metabolic disorder of uric acid includes excessive uric acid production and decreased uric acid excretion [26]. Causes of excess uric acid production include the intake of purine-rich foods, such as seafood and meat, and the increased concentrations or activities of intermediate metabolic enzymes of uric acid, such as xanthine oxidase [27]. Since more than 70% of uric acid in the human body is produced by metabolism, inhibiting the activities of metabolic enzymes can effectively inhibit the production of uric acid [28]. Therefore, xanthine oxidase inhibitors such as allopurinol, febuxostat, and topiroxostat are the drugs of choice for the clinical treatment of hyperuricemia and gout [29]. In addition, the main reason for the decrease in uric acid excretion is closely related to the insufficient renal excretion capacity. This is because the kidney is the main excretory organ of uric acid, and more than 2/3 of uric acid is excreted from the kidney [30]. Therefore, promoting the excretion of uric acid by the kidney by regulating the activities of uric acid transporters is an effective method to treat hyperuricemia and gout. Current studies have found that uric acid transport-related proteins mainly include uric acid reabsorption-related proteins and uric acid secretion-related proteins (Figure 2). Proteins related to uric acid reabsorption include URAT1, glucose transporter 9 (GLUT9), organic anion transporter 4 (OAT4), and organic anion transporter 10 [31]. Proteins related to uric acid secretion include organic anion transporter 1, organic anion transporter 2, organic anion transporter 3, sodium-dependent phosphate transport protein 1 (NPT1), sodium-dependent phosphate transport protein 4, ATP-binding cassette superfamily G2 (ABCG2), multidrug resistance protein 4 (MRP4), and urate transporter (UAT) [32]. Among these proteins, URAT1 is a highly valuable potential therapeutic target.

URAT1 is encoded by the SLC22A12 gene, which is located on chromosome 11q13, contains 10 exons and 9 introns, encodes 555 amino acids, and has 12 transmembrane domains [33]. URAT1, originally called the renal-specific transporter, is a member of the organic anion transporter family and the first protein to be involved in renal uric acid transport [34]. Figure 2 shows that URAT1 is located in the renal tubule epithelial cell apical membrane and mediates the exchange of uric acid in the lumen with inorganic and organic anions in the proximal tubular epithelial cells, thus reabsorbing uric acid from the lumen into epithelial cells [35]. Although URAT1 is not the only protein that mediates uric acid re-absorption, the importance of URAT1 is reflected in its strong transport capacity: approximately 90% of uric acid re-absorption is mediated by URAT1 [36]. Therefore, considering the important role of URAT1 in uric acid re-absorption, URAT1 inhibitors are considered highly effective and promising drugs for the treatment of hyperuricemia. As early as 2002, related studies explored the possibility and value of URAT1 as a target for reducing uric acid and first proposed the development of URAT1 inhibitors [37]. So far, researchers have developed a variety of URAT1 inhibitors, such as probenecid, benzbromarone, lesinurad, and dotinurad [12]. These drugs can effectively inhibit the reabsorption of uric acid by URAT1 and promote the excretion of uric acid, thus exerting a uric acid-lowering effect.

## 3. Natural Products with URAT1 Inhibitory Effects

Due to the great potential of URAT1 inhibitors in the treatment of hyperuricemia and gout, researchers have been exploring new URAT1 inhibitors [38]. As an important source of new drugs, natural products have received more and more attention for their inhibitory effects on URAT1 [39].  Table 1 summarizes the species, main sources, and inhibitory effects of URAT1 of these natural products. It can be seen that many of the natural products with URAT1 inhibition are flavonoids [45, 52, 53]. In addition, some terpenoids, alkaloids, coumarins, stilbenes, and steroids also show a URAT1 inhibitory effect [60, 66, 68, 74, 77]. However, most studies have focused on finding natural products with inhibition of URAT1 and have not explored their mechanisms toward URAT1 [63, 64]. Therefore, exploring the mechanism of action will be an important research direction in the future.

### 3.1. Flavonoids

**Figure 3:**
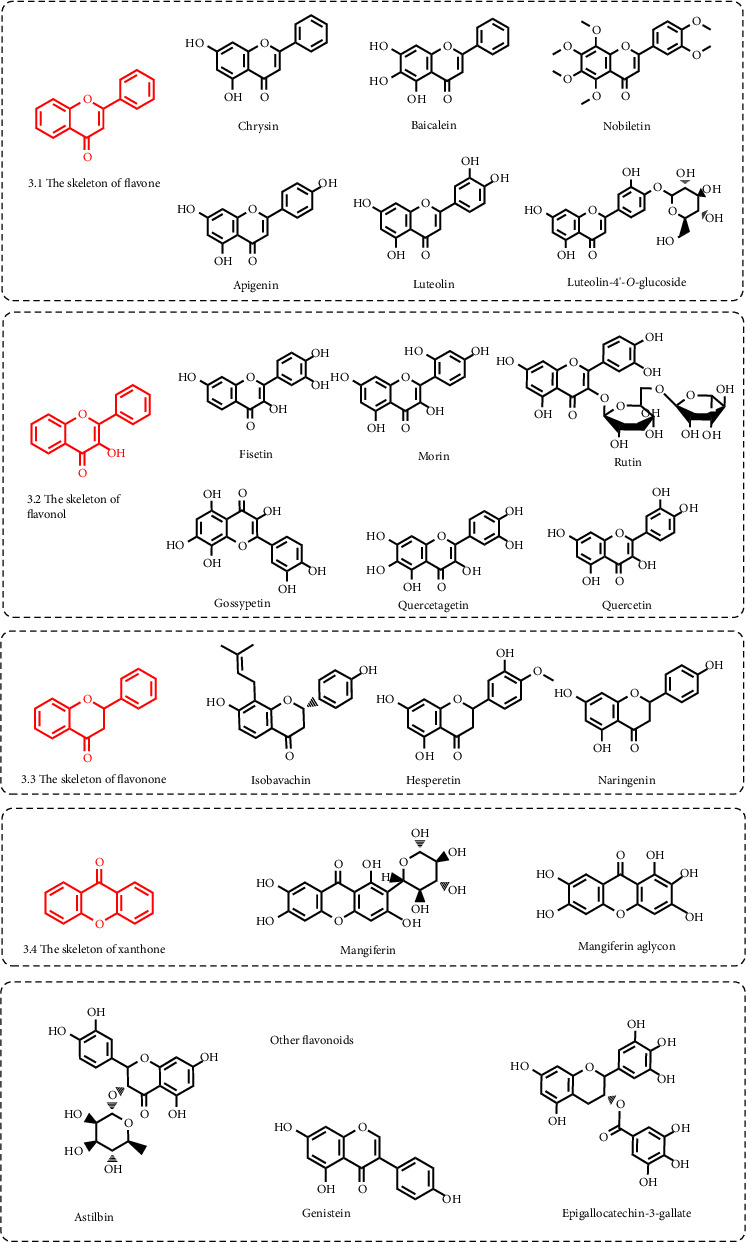
Structural formula of flavonoids with URAT1 inhibitory effect.

Flavonoids are a class of secondary plant metabolites widely present in a variety of plants and are the active components of many Chinese herbal medicines. Chemical structure generally refers to the connection of two benzene rings (ring A and ring B) through three carbon atoms to form the structure C_6_-C_3_-C_6_ [78]. Flavonoids contain many subclasses based on the connection position of the B and C rings as well as the degree of saturation, oxidation, and hydroxylation of the C ring [79]. Currently, studies have shown that many natural products with URAT1 inhibitory effects belong to flavonoids, and the subclasses include flavones, flavonols, flavanols, flavonones, flavanonols, isoflavones, and xanthones.  Figure 3 further summarizes the structural formulas of these flavonoids.

#### 3.1.1. Flavones

It can be seen in Figure 3 that flavones are characterized by containing a double bond between positions 2 and 3 and a ketone in position 4 of the C ring [80]. Currently, flavones with the inhibitory effect of URAT1 include chrysin, apigenin, baicalein, nobiletin, and luteolin. The structures of these flavones are very similar, except for nobiletin (the substituents are all methoxy). They have hydroxyl groups at positions 5 and 7 of the A ring, and the differences are reflected in the number of hydroxyl groups at positions 3, 4, and 5 of the B ring. Chrysin is mainly derived from propolis, blue passion flower, and honey [81]. In rats induced by high fructose corn syrup hyperuricemia, chrysin (50–150 mg/kg) could inhibit the expression of URAT1 and promote uric acid excretion [44]. The main sources of apigenin are the leafy herbs parsley and dried chamomile flowers [82]. Cellular experiments showed that apigenin (3.125–100 *μ*M/l) could inhibit cellular uptake of uric acid in HK-2 cells treated with uric acid by inhibiting URAT1 expression [42]. Li et al. found that apigenin (IC_50_ = 0.64 *μ*M/l) not only competitively inhibited URAT1 activity in vitro, but also (100 mg/kg) promoted uric acid excretion by inhibiting URAT1 activity in potassium oxonate-induced hyperuricemic nephropathy mice [43]. The main source of baicalein is the root of *Scutellaria baicalensis*. Baicalein (IC_50_ = 31.56 *μ*M/l) could non-competitively inhibit URAT1 activity in vitro and (200 mg/kg) improved renal urate excretion by inhibiting URAT1 expression in potassium oxonate-induced hyperuricemia mice. Protein docking analysis revealed that baicalein interacted with Ser35 and Phe241 of URAT1 [41]. Nobiletin is a highly methoxylated flavone compound, especially abundant in citrus [40]. Cell experiments showed that nobiletin (IC_50_ = 17.6 *μ*M/l) could inhibit URAT1 expression and uric acid uptake in 293A cells expressing URAT1 treated with uric acid [51]. Luteolin is widely found in fruits and vegetables [83]. The animal experiment showed that both luteolin (3–10 mg/kg) and luteolin-4′-*O*-glucoside (20–100 mg/kg) could inhibit URAT1 expression and promote uric acid excretion in potassium oxonate-induced hyperuricemia mice [45].

#### 3.1.2. Flavonols

It can be seen in Figure 3 that flavonols are characterized by containing a hydroxyl group at position 3 of the C ring [84]. Current studies show that six flavonols are promising as URAT1 inhibitors, which are gossypetin, quercetagetin, quercetin, fisetin, morin, and rutin. Cellular experiments showed that gossypetin (isolated from *Hibiscus sabdariffa* flowers), quercetagetin (isolated from tagetes flowers), and quercetin (widespread in vegetables and fruits) could inhibit URAT1 expression and uric acid uptake in 293A cells expressing URAT1, and the IC_50_ values were 31.3 *μ*M/l, 18.4 *μ*M/l, and 12.6 *μ*M/l, respectively [50, 85–87]. Rutin, also called rutoside, quercetin-3-rutinoside, and sophorin, is abundant in vegetables and fruits, such as passion flower, tea, apple, asparagus, blackberry, quince, cherry, and red plum [88]. Fisetin is also widely found in vegetables and fruits, such as strawberry, blueberry, apple, grape, persimmon, kiwi, and cucumber [89]. The source of morin is mainly Moraceae plants [90]. Animal studies have shown that fisetin (50–100 mg/kg), morin (10–40 mg/kg), and rutin (25–100 mg/kg) could inhibit URAT1 activity and promote uric acid excretion in potassium oxonate-induced hyperuricemia mice [46–49].

#### 3.1.3. Flavanols

Compared to flavonol, the structural characteristic of flavanol is that the C ring has no carbonyl group and the double bond at positions 2 and 3 is hydrogenated [91]. Flavanols are divided into flavan-3-ols and flavan-3,4-diols according to the position of the hydroxyl group in the C-ring. Current research has shown that only epigallocatechin-3-gallate, the main component of green tea polyphenols, has a URAT1 inhibitory effect [92]. As can be seen from the structure, epigallocatechin-3-gallate is an ester formed by epigallocatechin and gallic acid and belongs to the flavan-3-ols. The animal study showed that epigallocatechin-3-gallate (10–50 mg/kg) inhibited the expression of URAT1 and promoted uric acid excretion in hyperuricemia mice induced by potassium oxonate [53].

#### 3.1.4. Flavonones

It can be seen in Figure 3 that the structural characteristic of flavonone is that the double bond at positions 2 and 3 of the C ring is hydrogenated [93]. Current research shows that flavonones with the inhibitory effect of URAT1 include hesperetin, naringenin, and isobavachin [94]. Both hesperetin and naringenin derive mainly from citrus fruits such as oranges and lemons [95]. Isobavachins are derived from the seeds of *Psoralea corylifolia* L. [96]. Hesperetin (IC_50_ = 17.6 *μ*M/l) and naringenin (IC_50_ = 16.1 *μ*M/l) could inhibit URAT1 expression and uric acid uptake in URAT1-expressing 293A cells [51]. Isobavachin could also inhibit URAT1 expression and uric acid uptake in URAT1-expressing HEK293 cells (IC_50_ = 0.39 *μ*M/l) and promote uric acid excretion in potassium oxonate-induced hyperuricemia mice (10 mg/kg) [52].

#### 3.1.5. Flavanonols

It can be seen in Figure 3 that flavanonol is produced by hydrogenation of the double bond at positions 2 and 3 of the C ring of flavonol [97]. The current study showed that only astilbin, a flavanonol glucoside of *Smilax glabra*, has the inhibitory effect of URAT1 [98]. In potassium oxonate-induced hyperuricemia mice, astilbin (5–20 mg/kg) inhibited URAT1 expression and promoted the excretion of uric acid [54, 55].

#### 3.1.6. Isoflavones

Compared to flavone, the structural characteristic of isoflavone is that the B ring is attached to the 3-position of the C ring [99]. Current research has shown that only genistein derived from plants of Leguminoseae has the inhibitory effect of URAT1 [100]. In potassium oxonate-induced hyperuricemia mice, genistein (10–20 mg/kg) inhibited URAT1 expression and promoted uric acid excretion [56].

#### 3.1.7. Xanthones

Xanthones (dibenzo-*γ*-pyrones) constitute an important class of oxygenated heterocycles and occur as secondary metabolites in plants and microorganisms. Xanthones do not conform to the basic skeleton of C_6_-C_3_-C_6_, but are also classified as flavonoids due to their benzo *γ*-pyranone structure [101]. Current research has shown that xanthones with the inhibitory effect of URAT1 include mangiferin and mangiferin aglycon, and they mainly derive from *Mangifera indica* L. [102]. The animal experiment showed that mangiferin (1.5–24.0 mg/kg) and mangiferin aglycon (10–30 mg/kg) inhibited URAT1 expression and promoted uric acid excretion in potassium oxonate-induced hyperuricemia mice [57, 58].

### 3.2. Terpenoids

**Figure 4:**
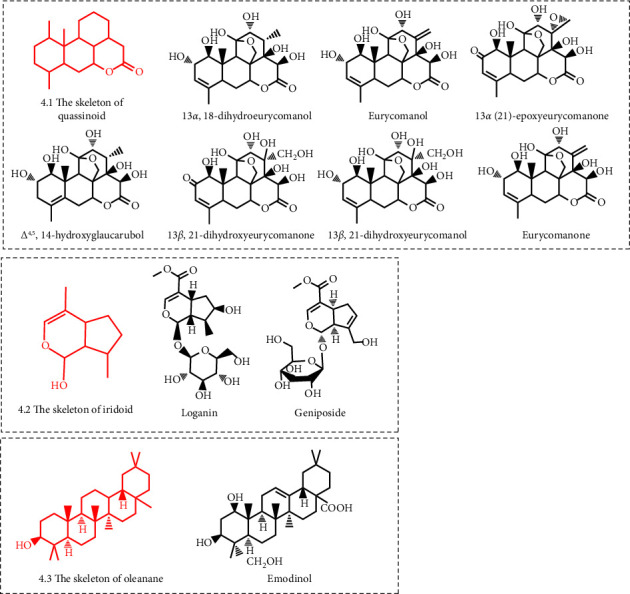
Structural formula of terpenes with URAT1 inhibitory effect.

Terpenoids consist of isoprene units and can be divided into hemiterpenes, monoterpenes, sesquiterpenes, diterpenes, disesquiterpenes, triterpenes, and polyterpenes according to the number of units containing isoprene [103]. The current study shows that terpenoids with an URAT1 inhibitory effect include monoterpenes and triterpenes (Figure 4).

Only two iridoids among monoterpenes show the URAT1 inhibitory effect, including loganin and geniposide. Loganin is a common iridoid glycoside derived from *Cornus officinalis* [104]. Geniposide is also an iridoid glycoside and an important active ingredient of *Gardenia jasminoides* [105]. Studies have shown that both loganin (20–40 mg/kg) and geniposide (100–200 mg/kg) could inhibit URAT1 activity and promote uric acid excretion in potassium oxonate-induced hyperuricemia mice [63, 65].

Triterpenoids with URAT1 inhibitory effect are mainly a series of quassinoids extracted from *Eurycoma longifolia*, including eurycomanol, eurycomanone, 13*β*,18-dihydroeurycomanol, Δ^4,5^,14-hydroxyglaucarubol, 13*β*,21-dihydroxyeurycomanol, 13*β*,21-dihydroxyeurycomanone, and 13*α*(21)-epoxyeurycomanone [66]. The structural differences of these quassinoids are reflected in the differences of the substituents of the 2 and 21 positions. The cellular experiment showed that these quassinoids (50 *μ*M/l) decreased urate uptake in HEK293T cells expressing URAT1 by inhibiting URAT1 activity [66]. Furthermore, emodinol, a triterpenoid extracted from *Elaeagus pungens*, also had the inhibitory effect of URAT1, which (25–100 mg/kg) inhibited the expression of URAT1 and promoted uric acid excretion in potassium oxonate-induced hyperuricemia mice [67, 106].

### 3.3. Coumarins

**Figure 5:**
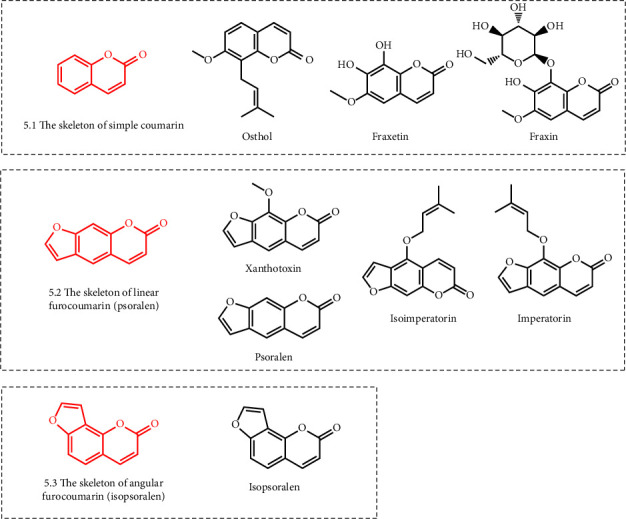
Structural formula of coumarins with the URAT1 inhibitory effect.

Coumarin is a general term for a class of natural compounds with benzo-*α*-pyrone core, which can be regarded as lactones formed by the dehydration of cis-o-hydroxycinnamic acid [107]. Currently, studies have shown that a variety of coumarins have an inhibitory effect on URAT1 (Figure 5), including psoralen and isopsoralen (extracted from *Cullen corylifolium*) [108], imperatorin and isoimperatorin (extracted from *Angelica dahurica and Angelica sinensis*) [109], xanthotoxin (extracted from *Zanthoxylum bungeanum*) [110], fraxetin and fraxin (extracted from *Fraxinus chinensis*) [111], and osthol (extracted from *Clinopodium megalanthum*) [112]. Osthol, fraxetin, and fraxin are simple coumarins characterized by the 7-position hydroxyl group not forming a furan or pyran ring with the 6- or 8-position isopentenyl group. Studies have shown that osthol (IC_50_ = 78.8 *μ*M/l) could non-competitively inhibit URAT1 activity in vitro, and both fraxetin and fraxin (20–40 mg/kg) could negatively regulate URAT1 expression in potassium oxonate-induced hyperuricemia mice [60, 61]. Xanthotoxin, psoralen, imperatorin, and isoimperatorin are linear furocoumarins formed by the condensation of the 7-position hydroxyl group with the 6-position isopentenyl group. Isopsoralen is an angular furocoumarin formed by condensation of the 7-position hydroxyl group with the 8-position isopentenyl group. Animal studies have shown that these furocoumarins (20–40 mg/kg) inhibited URAT1 activity and promoted uric acid excretion in mice with potassium oxonate-induced hyperuricemia nephropathy [59, 60].

### 3.4. Stilbenes

**Figure 6:**
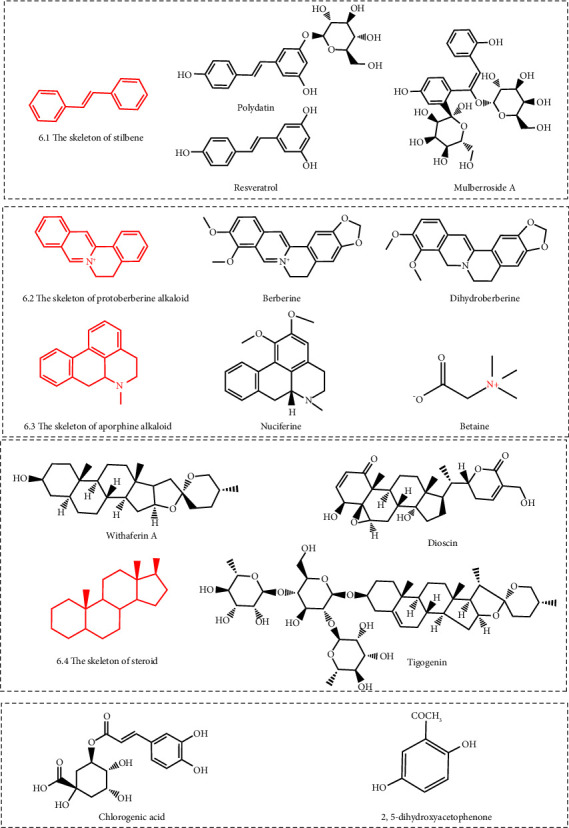
Structural formula of stilbenes, alkaloids, steroids, and other natural products with an URAT1 inhibitory effect.

Stilbenes refer to the general term of monomers with a 1,2-diphenylethylene skeleton and their polymers [113]. Current studies show that stilbenes with URAT1 inhibitory effects include resveratrol, polydatin, and mulberroside A (Figure 6). Resveratrol is a well-known stilbene compound present in grapes, soybeans, berries, pomegranate, and peanuts [114]. Animal experiments have shown that resveratrol (10–40 mg/kg) could promote uric acid excretion by inhibiting URAT1 activity in potassium oxonate-induced hyperuricemia mice. Furthermore, the researchers believed that this was related to inhibiting the activation of the inflammatory response, namely inhibiting the NLRP3 (NOD-like receptor family, pyrin domain-containing 3) inflammasome and TLR4 (toll-like receptor 4)/MyD88 (myeloid differentiation factor 88)/NF-*κ*B (nuclear factor-*κ*B) signaling pathway [62]. In addition to resveratrol, Chen et al. found that the resveratrol tetramer (20–60 mg/kg) could also inhibit URAT1 activity and promote uric acid excretion in mice with potassium oxonate induced hyperuricemia mice [77]. Polydatin and mulberroside A are two stilbene compounds with a very similar structure derived from *Polygonum cuspidatum* and *Morus alba* L., respectively [115, 116]. In potassium oxonate-induced hyperuricemia mice, polydatin (20–40 mg/kg) and mulberroside A (10–40 mg/kg) could down-regulate the expression of URAT1 in the kidney and promote uric acid excretion [63, 64].

### 3.5. Alkaloids

Alkaloids are a class of nitrogen-containing organic compounds derived primarily from plants [117]. Current studies show that alkaloids with URAT1 inhibition include betaine, nuciferine, berberine, and dihydroberberine (Figure 6). Betaine is a quaternary ammonium-type alkaloid derived from beet [118]. Nuciferine is an aporphine alkaloid extracted from *Nelumbo nucifera* [119]. Berberine and dihydroberberine are two isoquinoline alkaloids derived from *Coptis chinensis* and *Phellodendron chinense* [119, 120]. Animal experiments showed that betaine (5–40 mg/kg), nuciferine (5–40 mg/kg), berberine (6.25–25.0 mg/kg) and dihydroberberine (25–50 mg/kg) could inhibit URAT1 expression and promote the excretion of uric acid in potassium oxonate-induced hyperuricemia mice [68–71].

### 3.6. Steroids

Steroid is a general term for a large class of compounds with the basic skeleton structure of perhydrocyclopentano-phenanthrene [121]. Current studies have shown that steroids with an inhibitory effect of URAT1 include withaferin A, dioscin, and tigogenin (Figure 6). Dioscin is an isospirostane glycoside mainly extracted from the fenugreek plant [122]. Su et al. found that dioscin (319.22–1276.86 mg/kg) could negatively regulate URAT1 expression in potassium oxonate-induced hyperuricemia mice [72]. Tigogenin is a spirostane glycoside extracted from *Agave sisalana* [123]. Zhang et al. found that tigogenin (10–100 *μ*M/l) could decrease uric acid uptake in URAT1-expressing HCT116 cells by inhibiting URAT1 activity [73]. Withaferin A extracted from *Withania somnifera,* is a steroidal lactone [124]. In potassium oxonate-induced hyperuricemia mice, withaferin A (3–10 mg/kg) negatively regulated URAT1 expression in the kidney and promoted uric acid excretion [74].

### 3.7. Other Natural Products

Chlorogenic acid is an organic acid derived from honeysuckle [125]. In vitro research showed that chlorogenic acid (0.75 mmol/l) could inhibit uric acid reuptake in URAT1 expressing-HEK293T cells by inhibiting URAT1 expression [75]. Liang et al. isolated 2,5-dihydroxyacetophenone from *Ganoderma applanatum*, which (20–80 mg/kg) could inhibit URAT1 activity in mice induced by potassium oxonate hyperuricemia [76]. Li et al. isolated a pure polysaccharide ULP from *Ulva lactuca* consisting of rhamnose, glucuronic acid, galactose and xylose at a molar ratio of 32.75 : 22.83 : 1.07 : 6.46 with a molecular weight of 2.24 × 10^5^ Da. In potassium oxonate-induced hyperuricemia mice, ULP (10–50 mg/kg) inhibited the expression of URAT1 and promoted uric acid excretion [126].

## 4. Review and Speculation of the Structure-Activity Relationship

Currently, most studies have focused on finding natural products with inhibition of URAT1 and have not explored their activities and mechanisms toward URAT1. Therefore, in addition to exploring the mechanism of action, exploring the structure-activity relationship will be another important research direction in the future. By reviewing the few existing studies on the structure-activity relationship studies and analyzing common features of natural products with URAT1 inhibition, we speculate that the rigid ring structure and negative charge may be the keys for natural products to produce URAT1 inhibition. We hope that the analysis and speculation in this chapter can broaden the current understanding and trigger further interest in exploring the relationship between structure and URAT1 inhibition of natural products.

First, natural products may need to contain rigid structures. It can be found that among these natural products with URAT1 inhibition, almost all of them have a rigid ring structure. For example, one third of natural products with URAT1 inhibition are flavonoids. It can be seen from the structure of the flavonoid that two benzene rings connected to an oxygen-containing pyranyl group are a typical rigid plane molecular structure [127]. Polycyclic terpenoids, such as quassinoids, also contain unique rigid ring backbones [128]. In addition, other compounds also contain rigid ring structures, such as the benzene ring, the naphthalene ring, or the pyridine ring. Therefore, the presence of a rigid structure may be one of the elements that natural products must use to inhibit URAT1.

Second, natural products may require anions to act as URAT1 inhibitors. Wempe et al. evaluated the inhibitory effect of a series of (2-ethylbenzofuran-3-yl) (substituted-phenyl) methanone compounds on URAT1 activity in oocytes expressing hURAT1. The experimental data indicated that a potent hURAT1 inhibitor requires an anion (that is, a formal negative charge) to interact with the positively charged URAT1 binding pocket [129]. The C-ring of flavonoids is an electron-rich region with a strong negative charge. This partially explains the inhibitory activities of flavonoids in URAT1 [130]. Furthermore, most natural products with URAT1 inhibition also contain phenolic hydroxyl groups, so that these compounds can show acidity and generate anions [131]. However, alkaloids including betaine, nuciferine, berberine, and dihydroberberine also show inhibition of URAT1, so the presence of anions may not be a determinant of whether a natural product has inhibition of URAT1.

## 5. Conclusion and Prospects

In summary, current studies have shown that many natural products have a URAT1 inhibitory effect and are expected to be developed as URAT1 inhibitors, including flavonoids, terpenoids, alkaloids, coumarins, stilbenes, steroids, organic acids, and polysaccharides. The number of flavonoids is the largest among them, including many subtypes. Animal experiments have shown that these natural products can inhibit URAT1 activity in hyperuricemia mice and promote uric acid excretion. By reviewing the few existing studies on the structure-activity relationship studies and analyzing common features of natural products with URAT1 inhibition, we speculate that the rigid ring structure and negative charge may be the keys for natural products to produce URAT1 inhibition.

Although studies have confirmed that natural products are promising as URAT1 inhibitors, there are still some issues that need to be addressed in the future. First, the mechanism by which these natural products inhibit URAT1 is unclear. Therefore, more research is needed to explore the mechanism of action. Second, current research is still in the experimental study stage and it is necessary to carry out clinical research to further explore its therapeutic effects. Third, the relationship between structure and URAT1 inhibitory activity requires further investigation. In addition to the rigid ring structure and negative charge, what other structural features are essential for the URAT1 inhibitory effect of natural products? Fourth, structural modification is a common method to improve the therapeutic effect of drugs and reduce side effects. Therefore, structural modification based on clarifying the structure-activity relationship of natural products to improve the inhibitory activity of URAT1 may be a key research direction in the future.

## Figures and Tables

**Table 1:** Natural products with an URAT1 inhibitory effect.

Category	Name	Common or primary source	Cell lines/model	Dosage	Ref.
Flavones	Nobiletin	Citrus fruits	URAT1-expressing 293A cells	IC_50_ = 17.6 *μ*M/l	[40]
	Baicalein	*Scutellaria baicalensis*	URAT1 and potassium oxonate-induced hyperuricemia mice	IC_50_ = 31.56 *μ*M/l and 200 mg/kg, respectively	[41]
	Apigenin	The leafy herbs parsley and dried chamomile flowers	URAT1, URAT1-expressing HK-2 cells and hyperuricemia nephropathy mice	IC_50_ = 0.64 *μ*M/l, 3.125–100 *μ*M/l and 100 mg/kg	[42, 43]
	Chrysin	Propolis, blue passion flower, and honey	High fructose corn syrup-induced hyperuricemia rats	50–100 mg/kg	[44]
	Luteolin	Fruits and vegetable	Potassium oxonate-induced hyperuricemia mice	3–10 mg/kg	[45]
	Luteolin-4′-*O*-glucoside	Fruits and vegetable	Potassium oxonate-induced hyperuricemia mice	20–100 mg/kg	[45]

Flavonols	Fisetin	Vegetables and fruits	Potassium oxonate-induced hyperuricemia mice	50–100 mg/kg	[46]
	Morin	Plants and fruits of the Moraceae family	Potassium oxonate-induced hyperuricemia mice	10–40 mg/kg	[47, 48]
	Rutin	Vegetables and fruits	Potassium oxonate-induced hyperuricemia mice	25–100 mg/kg	[49]
	Gossypetin	Flowers of *Hibiscus sabdariffa*	URAT1-expressing 293A cells	IC_50_ = 31.3 *μ*M/l	[50]
	Quercetagetin	Tagetes flowers	URAT1-expressing 293A cells	IC_50_ = 18.4 *μ*M/l	[50]
	Quercetin	Vegetables and fruits	URAT1-expressing 293A cells	IC_50_ = 12.6 *μ*M/l	[50]

Flavonones	Naringenin	Citrus fruits	URAT1-expressing 293A cells	IC_50_ = 16.1 *μ*M/l	[51]
	Hesperetin	Citrus fruits	URAT1-expressing 293A cells	IC_50_ = 17.6 *μ*M/l	[51]
	Isobavachin	*Psoralea corylifolia* L.	URAT1-expressing HEK293 cells and hyperuricemia mice	IC_50_ = 0.39 *μ*M/l and 10 mg/kg, respectively	[52]
Flavanols	Epigallocatechin-3-gallate	Green tea	Potassium oxonate-induced hyperuricemia mice	10–50 mg/kg	[53]
Flavanonols	Astilbin	*Smilax glabra*	Potassium oxonate-induced hyperuricemia mice	5–20 mg/kg	[54, 55]
Isoflavones	Genistein	Leguminoseae plants	Potassium oxonate-induced hyperuricemia mice	10–20 mg/kg	[56].

Xanthones	Mangiferin	*Mangifera indica* L.	Potassium oxonate-induced hyperuricemia mice	1.5–24.0 mg/kg	[57]
	Mangiferin aglycon	*Mangifera indica* L.	Potassium oxonate-induced hyperuricemia mice	10–30 mg/kg	[58]

Coumarins	Psoralen	*Cullen corylifolium*	Potassium oxonate-induced hyperuricemia mice	20–40 mg/kg	[59]
	Isopsoralen	*Cullen corylifolium*	Potassium oxonate-induced hyperuricemia mice	20–40 mg/kg	[59]
	Imperatorin	*Angelica dahurica* and *Angelica sinensis*	Potassium oxonate-induced hyperuricemia mice	20–40 mg/kg	[59]
	Isoimperatorin	*Angelica dahurica* and *Angelica sinensis*	Potassium oxonate-induced hyperuricemia mice	20–40 mg/kg	[59]
	Xanthotoxin	*Zanthoxylum bungeanum*	Potassium oxonate-induced hyperuricemia mice	20–40 mg/kg	[59]
	Fraxetin	*Fraxinus chinensis*	Potassium oxonate-induced hyperuricemia mice	20–40 mg/kg	[60]
	Fraxin	*Fraxinus chinensis*	Potassium oxonate-induced hyperuricemia mice	20–40 mg/kg	[60]
	Osthol	*Clinopodium megalanthum*	URAT1 and potassium oxonate-induced hyperuricemia mice	IC_50_ = 78.8 *μ*M/l and 20–40 mg/kg	[61]

Stilbenes	Resveratrol	Grapes, soybeans, berries, pomegranate, and peanuts	Potassium oxonate-induced hyperuricemia mice	10–40 mg/kg	[62]
	Polydatin	*Polygonum cuspidatum*	Potassium oxonate-induced hyperuricemia mice	20–40 mg/kg	[63]
	Mulberroside A	*Morus alba* L.	Potassium oxonate-induced hyperuricemia mice	10–40 mg/kg	[64]

Terpenes	Loganin	*Cornus officinalis*	Potassium oxonate-induced hyperuricemia mice	20–40 mg/kg	[63]
	Geniposide	*Gardenia jasminoides*	Potassium oxonate-induced hyperuricemia mice	100–200 mg/kg	[65]
	13*β*, 18-dihydroeurycomanol	*Eurycoma longifolia*	URAT1-expressing HEK293T cells	50 *μ*M/l	[66]
	Δ^4,5^,14-hydroxyglaucarubol	*Eurycoma longifolia*	URAT1-expressing HEK293T cells	50 *μ*M/l	[66]
	13*β*, 21-dihydroxyeurycomanol	*Eurycoma longifolia*	URAT1-expressing HEK293T cells	50 *μ*M/l	[66]
	Eurycomanol	*Eurycoma longifolia*	URAT1-expressing HEK293T cells	50 *μ*M/l	[66]
	13*β*, 21-dihydroxyeurycomanone	*Eurycoma longifolia*	URAT1-expressing HEK293T cells	50 *μ*M/l	[66]
	13*α*21-epoxyeurycomanone	*Eurycoma longifolia*	URAT1-expressing HEK293T cells	50 *μ*M/l	[66]
	Emodinol	*Elaeagus pungens*	Potassium oxonate-induced hyperuricemia mice	25–100 mg/kg	[67]

Alkaloids	Betaine	Beet	Potassium oxonate-induced hyperuricemia mice	5–40 mg/kg	[68]
	Nuciferine	*Nelumbo nucifera*	Potassium oxonate-induced hyperuricemia mice	5–40 mg/kg	[69]
	Berberine	*Coptis chinensis* and *Phellodendron chinense*	Potassium oxonate-induced hyperuricemia mice	6.25–25.0 mg/kg	[70]
	Dihydroberberine	*Coptis chinensis* and *Phellodendron chinense*	Potassium oxonate-induced hyperuricemia mice	25–50 mg/kg	[71]

Steroids	Dioscin	Fenugreek plant	Potassium oxonate-induced hyperuricemia mice	319.22–1276.86 mg/kg	[72]
	Tigogenin	*Agave sisalana*	URAT1-expressing HCT116 cells	10–100 *μ*M/l	[73]
	Withaferin A	*Withania somnifera*	Potassium oxonate-induced hyperuricemia mice	3–10 mg/kg	[74]
Phenolic acids	Chlorogenic acid	Honeysuckle	Potassium oxonate-induced hyperuricemia mice	0.75 mmol/l	[75]
Acetophenone	2,5-Dihydroxyacetophenone	*Ganoderma applanatum*	Potassium oxonate-induced hyperuricemia mice	20–80 mg/kg	[76]

## Data Availability

Data sharing are not applicable to this article, as no new data were created or analyzed in this study.
